# Design of a smart hydroponics monitoring system using an ESP32 microcontroller and the Internet of Things

**DOI:** 10.1016/j.mex.2023.102401

**Published:** 2023-09-25

**Authors:** Anees Abu Sneineh, Arafat A.A. Shabaneh

**Affiliations:** a Department of Electrical Engineering, Faculty of Engineering and Technology, Palestine Technical University – Kadoorie (PTUK), Yafa Street, Tulkarm, Palestine; b Department of Telecommunication Technology Engineering, Faculty of Engineering and Technology, Palestine Technical University – Kadoorie (PTUK), Yafa Street, Tulkarm, Palestine

**Keywords:** monitoring Hydroponics system using IoT, Monitoring hydroponics, Blynk app, IoT, TDS sensor, pH sensor, ESP32

## Abstract

This paper presents the design and construction of a hydroponics monitoring system that can collect parameters of hydroponic systems, such as temperature, water limit, pH level, and nutrient levels. The monitoring system was developed using an ESP32 microcontroller and several sensors, including total dissolved solids (TDS), pH, water level, and temperature sensors. The ESP32 microcontroller gathers and processes data from the sensors to automatically activate the water or salt pump and drain the necessary materials into the hydroponic system's plant basin. The user can then view the hydroponic parameters through the Blynk application on a smartphone. The user can also activate the pumps for water, nutrients, or salt using the application's interface on a smartphone, or the ESP32 microcontroller can activate them automatically if the parameter values deviate from the required values. The monitoring hydroponics system and IoT interface were successfully built and implemented. The experiments were compiled, and the data gathered and discussed.

• An ESP32 microcontroller with TDS, pH, water level, and temperature sensors was used to build the hydroponic monitoring system.
• The ESP32 automatically collects and evaluates sensor data in order to drain water nutrients, or salt into the plant basin of the hydroponic system as necessary.
• The user can also check the parameters of the hydroponic system and, if necessary, run the pumps for water, fertilizers, or salt using his smartphone through the Blynk IoT app.

## Graphical abstract

**Figure:**
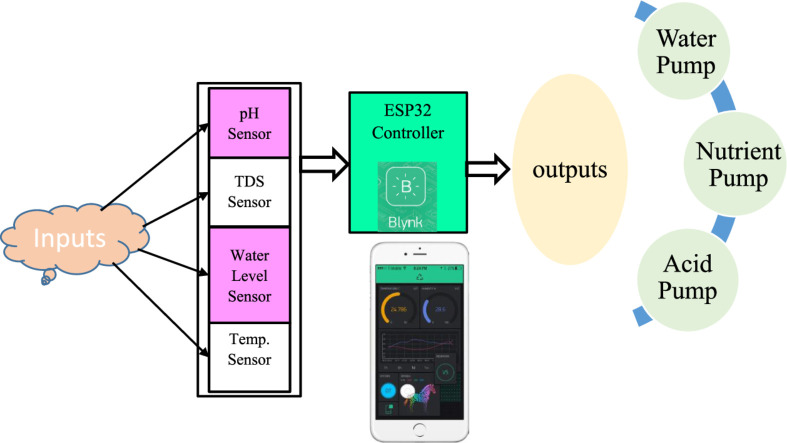

Specifications Table

**Table:** 

Subject area:	Engineering
More specific subject area:	Electronics engineering control applied in agriculture
Name of your method:	monitoring Hydroponics system using IoT
Name and reference of original method:	NA
Resource availability:	NA

## Introduction

Hydroponics is a technique of using water and fertilizer solutions as the growing medium, increasing productivity through monitoring of environmental conditions compared to traditional agricultural methods [1]. In a hydroponic system, plants are kept in tubs, and their roots float in nutrient-rich liquid, allowing them to develop rapidly and become a mass. Hydroponics, which translates to “water work”, comes from the Greek terms hydro, which means “water”, and ponos, which means “labor” [2]. This type of irrigation is frequently used in areas where the soil is not fertile enough to support crop production [3].

The problem that this study attempts to solve is how to reduce farmers' efforts in checking the elements that the plant needs without the requirement for farmer intervention. The hydroponics monitoring systems using Internet of Things (IoT) can be employed to reduce losses, optimize efficiency, increase productivity, and lessen the time and effort required. Monitoring the humidity, temperature, salinity, pH, oxygen, and nutritional levels of plants is an important aspect of farming. Smart systems are employed for their convenience, precision, and efficiency brought about by technological developments [4,5]. This technique has been used to grow fruits properly and has produced good results for larger plants, particularly lettuce, cucumbers, and tomatoes [6].

The Internet of Things (IoT) is a network of connected computers with mechanical and smart machinery, objects, living creatures, and people. These devices have identifiers and the ability to communicate information with one another and computers without the need for direct human or computer involvement. They can link and interact with others through the web, be automatically evaluated and controlled, and are packed with tools, online networks, and other equipment like sensors. The motivation of the researchers is to apply IoT technology to all areas of daily life and take advantage of these benefits [7], [8], [9].

The proposed system is novel in that it has the following contributions:

• Farmers can use sensor integration for data-based decision making because the system delivers accurate and continuous measurements.
• Farmers can monitor and change environmental parameters remotely because these systems include automation and control components.
• Farmers can evaluate the effectiveness of the hydroponic system because these systems examine the huge quantities of sensor data they receive.
• Farmers can prevent crop loss because the systems may send alarms when specific parameters stray from the appropriate range.
• The systems integrate with other technologies like IoT, are flexible and can be adapted to the particular needs of various crops and growing conditions, and contribute to resource optimization.

## Literature review

Hydroponics system uses nutrient-rich water as the growing medium for plants. The use of hydroponic systems in agricultural technology has grown significantly. It has the potential to partially replace conventional soil-based growth methods in global food production [10]. One benefit of hydroponic growing systems is the ability to control environmental factors to maximize production in vertical gardens to constrained spaces. Other benefits include reducing water waste through recirculation, growing crops in controlled environments (such as monitoring nutrition, plant pests, and other aspects necessary for optimal growth of plants), and the ability to control circumstances to increase the output of vertical gardens in limited spaces [11].

Kularbphyttong et al. [12] developed a mechanism for controlling plant growth. This system can regulate essential environmental elements that affect a plant's growth, such as temperature, humidity, and water. The application system automatically blends the chosen solution to determine the correct amount, collects data on the quantity of solution mixed during planting, and may be used to assess the cost of producing vegetables and determine the profitability of each produce to help with growth decisions.

Huo et al. [13] explored the influence of microalgae on vegetable growth and evaluated the nutrient removal in the greenhouse for three kinds of vegetables produced hydroponically in greenhouses using nitrate-rich synthetic wastewater. The results indicated that most vegetable types produced more as a response to the use of microalgae as a technique of sustainable production system.

Puno et al. [14] developed a hydroponics system that combines fuzzy logic control and nutrient film technology. Several crops can be grown within a limited space using this technique. The main parameters for crop survival were monitored, and fuzzy logic was used to drive the pumps for the tanks holding fresh water and nutrient concentrates. The drains of the mixing tanks were also monitored using data from sensors that detected electrical conductivity, pH, and water levels in the tank.

Ramos et al. [15] developed a hydroponics system with a nutrient film technique to investigate and create algorithms that will enable effective water recirculation, resulting in electricity savings of about 40% compared to conventional systems.

## Hardware implementation

The main parts of the proposed monitoring hydroponic system are described as follows.

### ESP32 microcontroller

**Fig. 1:**
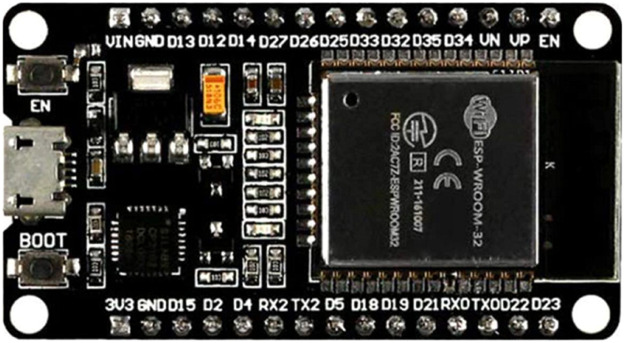
ESP32 microcontroller.

An ESP32 microcontroller with 38 pins, digital analog convertor (DAC), analog digital convertor (ADC), and built-in Wi-Fi connectivity was used. It is designed to focus on IoT applications. Fig. 1 shows the ESP32 microcontroller and its components. The ESP32 microcontroller was used as the monitoring device for the hydroponics process and controls the water, salt, pH, temperature, and humidity levels. All the data collected by the sensors are processed inside the ESP32 microcontroller before being sent through Wi-Fi to a database to be saved for further use.

### Total dissolved solids sensor

**Fig. 2:**
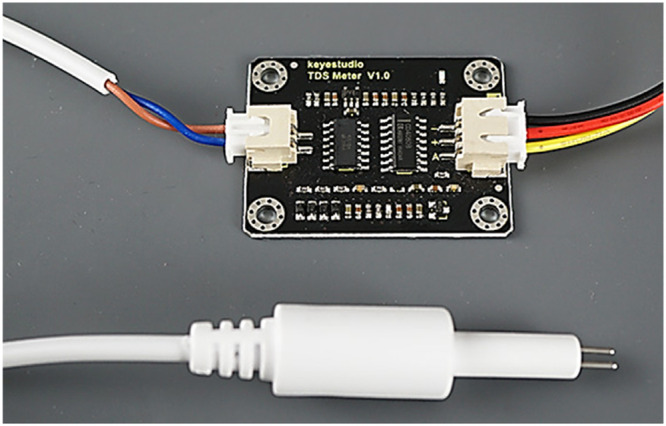
The TDS sensor.

The total dissolved solids (TDS) sensor in a hydroponic system is used to gage the concentration of soluble solids in the nutrient solution and expressed as electrical conductivity (EC). It can estimate how much salt dissolves in a liter of water in milligrams by the value of total part per million (PPM), which reflects how pure the water is to a particular level with an accuracy of ± 2% of 999 ppm TDS [16]. The TDS probe and printed board are shown in Fig. 2. Measurements during prolonged submersion in water are possible because the probe is waterproof [17].

### DS18B20 temperature sensor

**Fig. 3:**
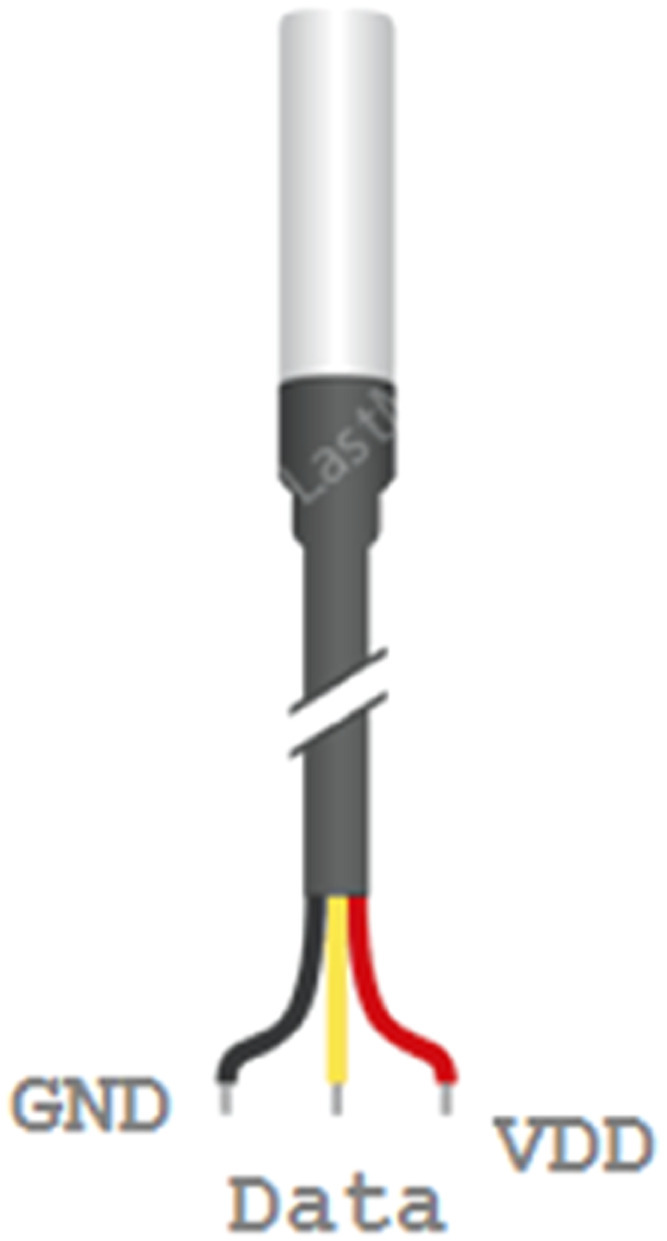
DS18B20 temperature sensor.

This sensor can measure temperatures from −55 °C to 125 °C with an accuracy of ±0.5 °C. ESP32 determines and records the signals specific to a particular protocol on its data pin to read the temperature from the sensor [18], [19], [20]. Fig. 3 shows the diagram of the DS18B20 temperature sensor.

### pH sensor

**Fig. 4:**
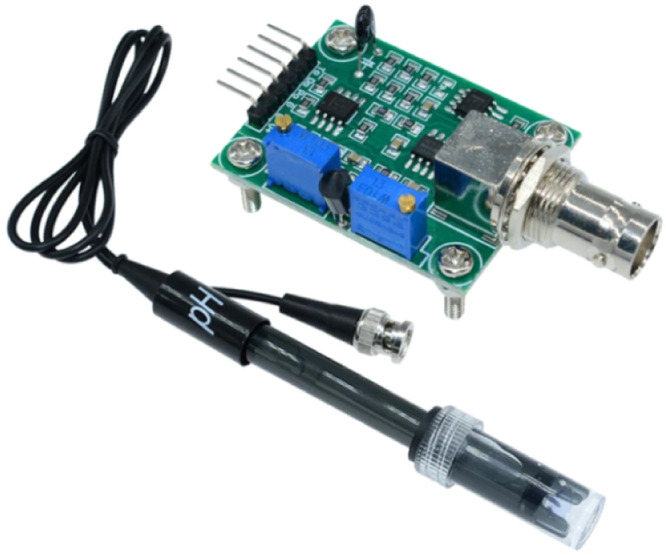
pH sensor.

This sensor identifies whether a substance or solution is basic, acidic, or neutral. The sensor can detect pH levels between 0 and 14, with an accuracy of approximately 0.1 pH. Plants require water with a pH between 6.5 and 7; thus, the nutrient solution's pH was maintained in this range [21]. Fig. 4 shows the applied pH sensor in the hydroponic systems.

### Blynk IoT application

A monitoring system using an ESP32 microcontroller and the Blynk IoT application was developed to evaluate the status of plants in a hydroponics system. Sensors collect the data, a microcontroller processes it, and the data are then transmitted to the Blynk server to be saved for further use. The user can employ that information to evaluate a plant's requirements and remotely activate the water, nutrient, or acid pump connected to the hydroponics system using the Blynk app.

**Fig. 5:**
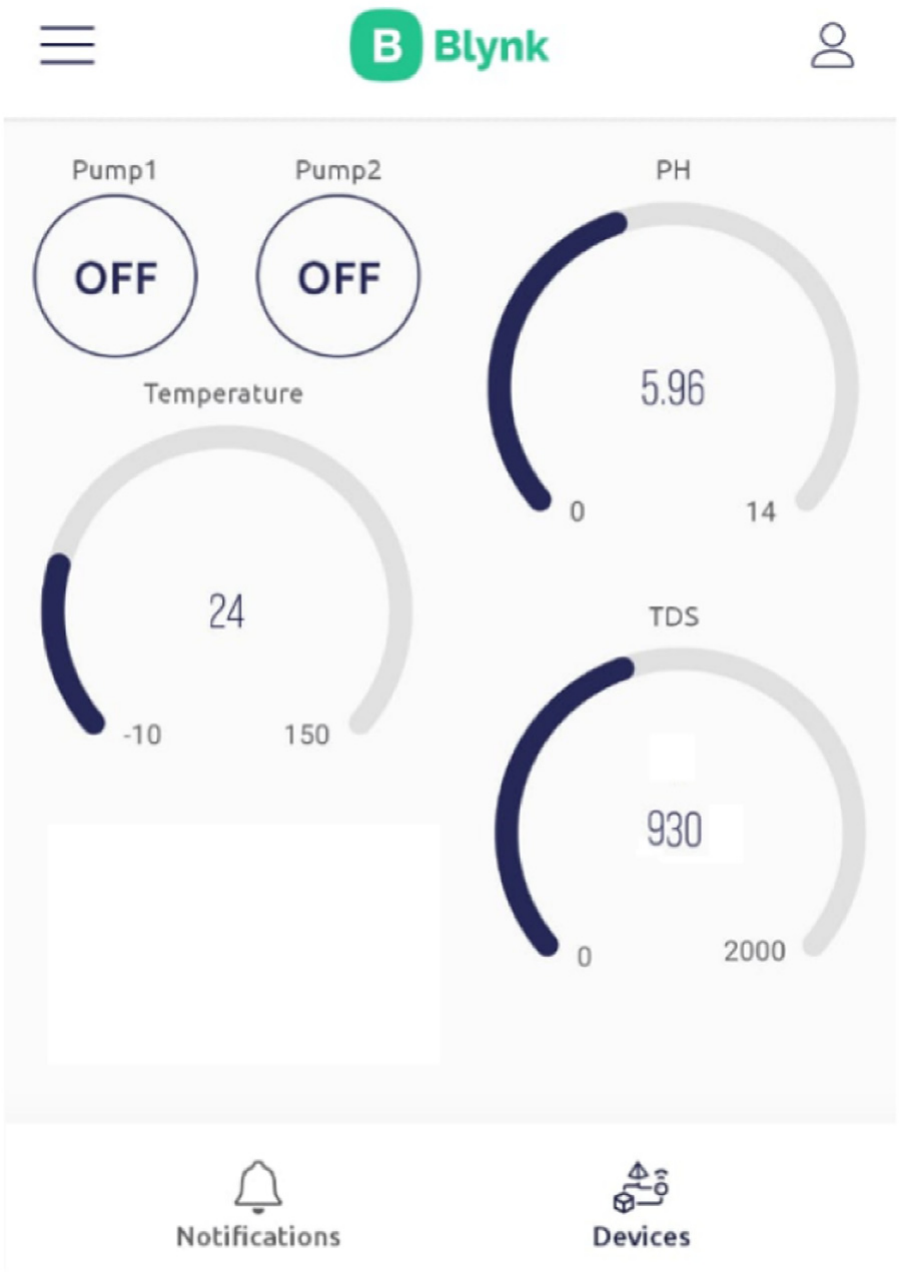
Blynk IoT application shortcut.

The Internet of Things (IoT) is a technology that enables any device to be controlled remotely [22]. The Blynk IoT application is an Android app that can be linked to Wi-Fi. The first step is to install the application and its libraries on a smartphone and the ESP32 microcontroller. After creating a new account on the Blynk server through an e-mail, the smartphone can add a new device and activate the connection with the microcontroller linked to the Wi-Fi. The interface can be designed according to the requirement of the system from the libraries of the Blynk app, which has more than 40 ready-to-use UI elements such as buttons, sliders, etc. After the design is completed, the application is linked to Wi-Fi. Fig. 5 shows the shortcut of the final Blynk IoT app. Using the Blynk IoT app, the user can view the hydroponics parameters shown in Fig. 5. If parameter values deviate from the required levels, the ESP32 microcontroller can automatically activate the salt or water pumps, or the user can manually activate them via the app's interface on a smartphone.

## Methodology

**Fig. 6:**
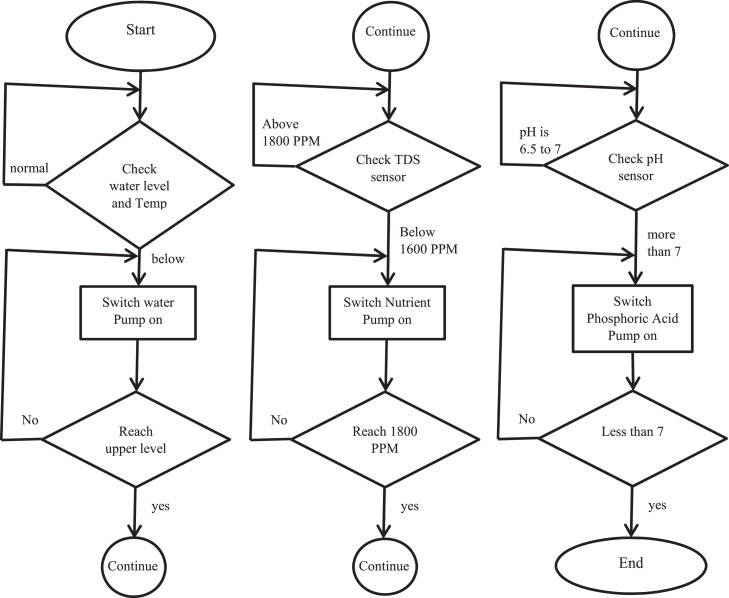
Flowcharts of the system.

The developed hydroponics monitoring system is implemented in the field at the Palestine Technical University- Kadoorie- Tulkarm- Palestine. The proposed design has three main tanks: the first tank contains fresh water mixed with fertilizer solution, the second tank contains the mixed potassium, phosphate, and nitrogen solution and the third tank is used to store the phosphoric acid solution. The sensors employed in the developed hydroponics monitoring system are water level, temperature, TDS, and pH. These sensors are mounted in the basin. The water level sensor transmits the water level record to the ESP32 microcontroller. If the water level drops below the predetermined level, the ESP32 microcontroller receives data from the sensor and instructs the water pump to compensate. Meanwhile, the temperature sensor monitors the water temperature to prevent adverse consequences to the plants should the temperature drop to below 17 °C. The TDS sensor measures the salt level in the water in the basin and transfers the recorded data to the ESP32 microcontroller. Thus, when the plant consumes a specific amount of salt, and the water's salt concentration EC falls below 1600 PPM, the ESP32 microcontroller receives information from the TDS sensor and instructs the salt pump to compensate for the salt loss through a particular concentration from the second tank. Fig. 6 demonstrates the main flowchart of the proposed hydroponic monitoring system.

**Fig. 7:**
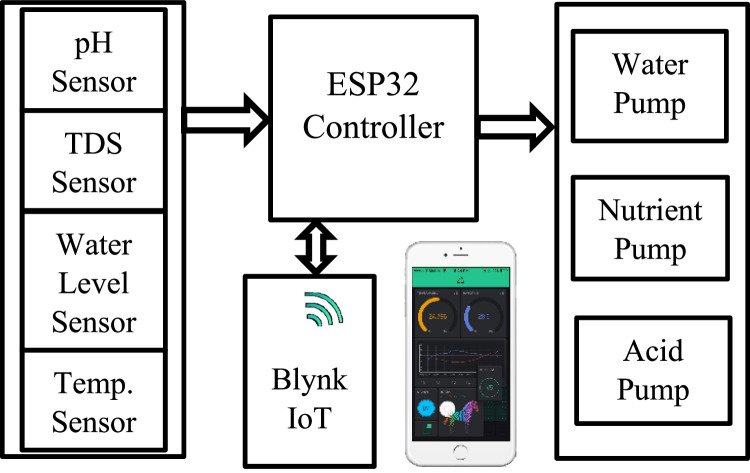
System block diagram.

The pH sensor measures the pH level of water in the cultivated basin and directly sends the data to the ESP32 microcontroller. Conventionally, plants require a pH degree of 6.5 to 7 in the hydroponics-cultivated basin. Therefore, if the pH rises above 7°, the ESP32 microcontroller will instruct the acid pump to add phosphoric acid to reduce the acidity to a reasonable level. Fig. 7 shows the block diagram of the main structure of the developed monitoring hydroponics system.

As mentioned previously, the sensors collect high precision real-time data on the environment of the hydroponics system and send it to the ESP32 microcontroller. The ESP32 microcontroller analyzes the data, turns on the suitable pump, and connects to the smartphone as IoT device through Wi-Fi. The mobile application and the ESP32 microcontroller communicate regularly with each other through IoT protocols, providing secure and efficient transportation of data between the mobile application and the IoT devices. Using a mobile application, the user provides instructions to the IoT devices to modify the relevant hydroponic environment parameters, such as the nutrient solution, water level, pH level, or temperature, within certain limits. Users can remotely check on the status of the hydroponic system with the help of the smartphone application, which continuously receives and shows real-time data from the IoT devices.

## Results and discussion

**Fig. 8:**
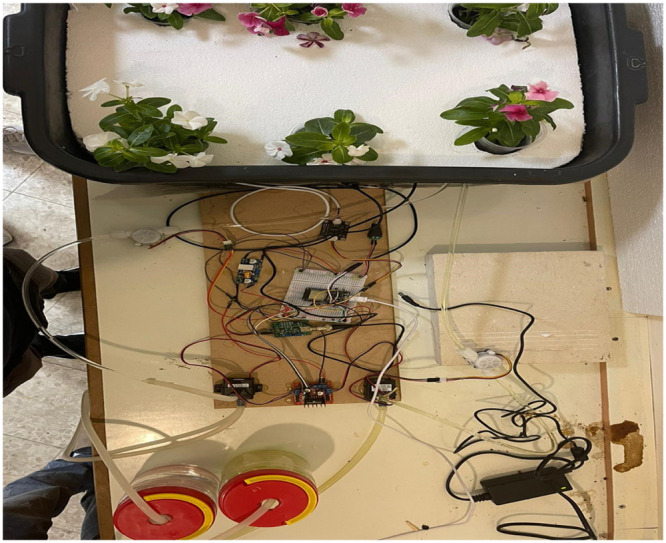
Prototype of the project.

The completed prototype of the hydroponic monitoring system is shown in Fig. 8. The ESP 32 controller and smartphone were programmed with the Blynk IoT application, and connectivity between the ESP 32 controller and the smartphone was established. The shortcut of the final Blynk IoT app is shown in Fig. 5. The experiments proved that monitoring of the hydroponics system was successfully carried out. For two weeks, daily statistics data from the Blynk app were gathered at the same time. Most of the results, except for temperature, were constant, as shown in Table 1, because the system compensates for changes in water, salt, or nutrition liquid.

**Table 1:** Daily data gathered from the Blynk app.

Day Parameter	Day 1	Day 2	Day 3	Day 4	Day 5	Day 6	Day 7	Day 8	Day 9	Day 10	Day 11
pH	6.8	6.8	6.9	6.8	6.8	6.9	6.8	6.8	6.8	6.8	6.9
EC (ppm)	1780	1778	1781	1778	1779	1777	1782	1778	1778	1782	1777
Temp. (°C)	20.5	20.5	21	21.5	22	21.5	20.4	20.5	20.9	20.8	21.2

The system was activated, and weekly reading was taken at the same time of day over five weeks to evaluate the monitoring system and generate realistic varying data accurately. Table 2 presents a sample of these results from the experiments completed under the previous conditions.

**Table 2:** Weekly reading data gathered from the Blynk app.

Week Parameter	1st week	2nd week	3rd week	4th week	5th week
pH	7.498	7.19	6.45	6.31	6.43
EC (ppm)	935.8	1078	1309	1444	1718
Temperature (°C)	17.96	21.4	28	31	34.4

The daily and weekly measurements indicate that the hydroponics’ parameters have been changing rapidly since the plants consumed the water and nutrient solution. Thus, daily monitoring of the system must be applied to fix the values of parameters.

The benefit of the measured values is that the plants’ variables can be stabilized, avoiding crop loss and increasing productivity. For example, an increase in the pH of more than 7.5 or an increase in the EC of more than 1800 ppm prevents the plant from absorbing nutrients, resulting in the plant's death and crop loss.

**Fig. 9:**
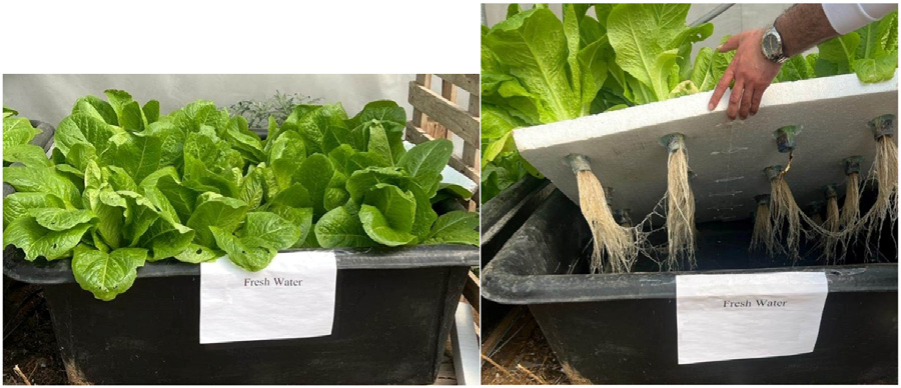
Hydroponic system monitored by the proposed system.

This system can be used with any plant that can be grown hydroponically, and the programming can be modified for various parameters, including the pH and salt percentage that suit the plant's needs. Fig. 9 shows an additional option to apply the proposed system to monitor a hydroponic lettuce plant system.

Because the average daily temperature in Palestine is between 17 and 33°, the temperature around the plant rarely needs to be adjusted. However, if this system were to be used in a country with a different average daily temperature, the programming could be modified by adding heaters or fans to increase or decrease the temperature to a suitable level for plants.

Table 3 shows a comparison of several studies from previous work. The table shows that the average pH ranges from 6.01 to 7.5, with our pH being about 6.8 with daily readings and possibly reaching 7.5 with weekly readings. Additionally, the EC values range varies from 1098.29 to 1448.84 ppm, while our EC is 1296.96 ppm with daily readings and may reach up to 1779 ppm with weekly readings. The studies indicated that different kinds of plants or environmental situations could change the parameters of a hydroponic system. The variations in the observed pH and EC values may be due to environmental variables. Additionally, because different kinds of plants have differing nutritional needs, absorption of nutrients might change the pH and EC values. The reliability and precision of the monitoring equipment employed to determine pH and EC may also influence the results. For the best nutrition availability for plants, pH and EC levels can be adjusted more precisely by using real-time data from control and monitoring systems. Application of new monitoring equipment and enhancement of the environmental parameters could yield better regulated pH and EC levels that could increase plant growth and production.

**Table 3:** Comparison data with previous work.

Refs. number	Controller name	Average pH	Average water temp .( °C)	Average EC (ppm)	Communication technology
[23]	Arduino Mega 2560	6.60	32.28	1448.84	IoT, Wi-Fi
[24]	Arduino, Raspberry Pi3	7.5	31		IoT
[25]	Arduino, Raspberry Pi 4	6.01	25.06	1344	spectroscopic IoT sensor
[26]	Esp8266				NodeRed IoT, Wi-Fi
[27]	Arduino Uno, GSM Shield	6.11	29	1098.29	Wi-Fi, xively server
[28]	Arduino Mega, ESP8266	6.2	28.9	1429	Ubidots Cloud
Proposed work	ESP32	6.8	26.55	1779	Blynk IoT apps, Wi-Fi, smartphone

## Conclusions

A hydroponics monitoring system is designed and fabricated based on an ESP32 microcontroller and integrated with Wi-Fi technologies as an interface to assist farmers or owners of hydroponic farming systems. Based on the data collected from sensors in the plant basin, the ESP32 microcontroller automatically activates the water, phosphoric acid, and fertilizer pumps (N, P, and K). TDS, pH, water level, and temperature sensors are also integrated into the developed hydroponics monitoring system. Temperature, water limit, pH level, and EC levels are measurable parameters in a hydroponics monitoring system. During the hydroponics period of lettuce crop, the average pH meter was maintained at around 7° while the EC level was below 1800 ppm, and the temperature was less than 27 °C. The Wi-Fi interface on the hydroponic monitoring and management system enables the control and monitoring of the hydroponics parameters of agricultural systems using the Blynk mobile application. This system can be easily obtained on the market at a relatively affordable price, and it can be implemented in the hydroponics system to enhance the plant growth. In this study, the monitoring of hydroponics system and IoT interface were effectively developed and implemented.

## CRediT authorship contribution statement

**Anees Abu Sneineh:** Conceptualization, Methodology, Data curation, Validation, Investigation, Software, Writing – original draft, Writing – review & editing. **Arafat A.A. Shabaneh:** Conceptualization, Methodology, Data curation, Validation, Investigation.

## Declaration of Competing Interest

The authors declare that they have no known competing financial interests or personal relationships that could have appeared to influence the work reported in this paper.

## Data Availability

The data that has been used is confidential.
